# Veterinary Diagnostic Practice and the Use of Rapid Tests in Antimicrobial Stewardship on UK Livestock Farms

**DOI:** 10.3389/fvets.2020.569545

**Published:** 2020-10-15

**Authors:** Henry Buller, Katie Adam, Alison Bard, Ann Bruce, Kin Wing (Ray) Chan, Stephen Hinchliffe, Lisa Morgans, Gwen Rees, Kristen K. Reyher

**Affiliations:** ^1^Geography Department, College of Life and Environmental Sciences, University of Exeter, Exeter, United Kingdom; ^2^INNOGEN Institute, Science, Technology and Innovation Studies, University of Edinburgh, Edinburgh, United Kingdom; ^3^School of Veterinary Sciences, University of Bristol, Bristol, United Kingdom

**Keywords:** antimicrobial use, livestock farms, veterinarians, rapid diagnostic tests, diagnostic practice

## Abstract

In this paper we consider the shifting role, practice and context of veterinary diagnosis in addressing concerns over what is, in the context of the growing threat of antimicrobial resistance, considered unnecessary or excessive antimicrobial medicine use in UK livestock farms. With increasing policy and regulatory interest in diagnostic practices and technologies, coupled with an expanding focus on the development and deployment of new rapid and point-of-care on-farm diagnostic testing, this paper investigates current diagnostic practices amongst veterinarians working on dairy, pig and poultry farms in Great Britain (England, Wales, and Scotland) and, more specifically, veterinarians' use and perceptions of new and emerging rapid and point-of-care diagnostic tests. Drawing on a series of 30 semi-structured interviews with farm animal veterinary professionals across the three sectors, this paper examines the manner in which such tests are both used and anticipated in clinical farm animal veterinary practice and the possible impact rapid test technologies might have on broader farm animal health management and disease control. Analysis of the transcribed interviews reveals a number of complexities around the use of rapid and point-of-care diagnostic tests. The relative rapidity and simplification of such tests, facilitating immediate treatment responses, is held in balance against both the accuracy and the more detailed and documented procedures of established laboratory testing routes. In situations of multifaceted on-farm etiologies, respondents maintained that rapid tests may offer restricted diagnostic capabilities, though in other situations they were found to offer ready confirmation of disease presence. A third complexity arising from the growth of rapid and point-of-care testing and revealed in this study relates to the shifting distribution of responsibilities in animal health care within contemporary food chains. The growing availability of rapid and point-of-care tests effectively diversifies the range of diagnostic actors with consequences for the flow of diagnostic and disease information. The veterinarians in this study identified areas where new rapid and point-of-care tests would be of particular value to them in their clinical practice particularly in addressing concerns over inappropriate antimicrobial use in animal treatment. However, despite the considerable policy advocacy on rapid and point-of-care tests as key tools in shifting diagnostic practice and reducing unnecessary antimicrobial use, veterinarians in this study, while recognizing the potential future role of such tools and technologies, nonetheless viewed diagnostic practice as a far more complex process for which rapid tests might constitute only a part.

## Introduction

In 2015, the UK's Review of Antimicrobial Resistance, originally commissioned in 2014 to investigate the emerging issue of antimicrobial resistance and propose workable solutions, released two reports. The first of these, published in October of that year, looked specifically at the role of rapid or point-of-care diagnostics in human medicine in reducing unnecessary antimicrobial use (1). The second, appearing two months later and entitled “Antimicrobials in Agriculture and the Environment” (2), sought to identify means to reduce the use of antimicrobials in livestock systems. Building on the earlier document, this second report also highlighted the potential role that rapid and point-of-care diagnostic tools might also play in reducing antimicrobial use in animal treatment. These two documents, followed by the Review's final report (3)–also known as the O'Neill report after the Review's Chairman–have since become key statements in informing subsequent responses of Government (4), NGOs (5), and industry (6) to the need to reduce antimicrobial use in agriculture and to the potential role of rapid and point-of-care diagnostic tests (which we define, for both terms, following Abuelo and Alves-Nores [(7), p. 293], as diagnostic tests “performed at or near the site of care,” producing a result in a short period of time on the farm thereby allowing a faster decision to be made about treatment) in that reduction. The UK Government, under its current Five-Year Action Plan for Antimicrobial Resistance, thereby committed to:

Explore, in collaboration with industry, options to develop rapid and reliable diagnostic tools to inform veterinarians' prescribing decisions; and promote the uptake of these tools (4).

The emphasis placed here on new, rapid and point-of-care veterinary diagnostic procedures and practices comes at a critical time within the development of both livestock agriculture and the veterinary profession. The former is becoming increasingly data-rich, through food chain monitoring, “smart” technologies and assurance processes leading to improved surveillance of animal health. The latter, meanwhile, with innovations in diagnostic and treatment technologies, is nevertheless adapting to different roles and structures as corporate veterinary practices become ever more present across the UK veterinary landscape (8–10) and as the responsibility of the farm animal veterinarian expands from the traditional role of animal “doctor” to include broader terrains of environmental and health planning as well as management within an increasingly vertically integrated agro-food sector (11–13). It is against this background that the role, the practice and the technologies of veterinary diagnosis are increasingly being discussed and debated both within the veterinary profession and beyond. In this paper, drawn from an ongoing social science-led research project (Diagnostic Innovation in Agriculture [DIAL], www.dialamr.com) into the broader function of diagnostics and diagnostic practice in farm animal treatment, we first consider the shifting place of veterinary diagnosis and stewardship with respect to antimicrobial use in livestock production. Taking insights and analysis from a recent qualitative study of diagnostic methods, tools and point-of-care technologies used by farm animal veterinarians in England, Scotland, and Wales, we then go on to investigate and report on how broader advocacy of rapid and point-of-care solutions becomes translated into current veterinary practices and the potential impact such diagnostic test technologies might have on farm health management and disease control. In doing so, this paper identifies a series of drivers and impacts of change which, we argue, have a potentially significant influence upon the role and place of farm animal veterinarians in contemporary agro-food systems.

## Diagnosis and Antimicrobial Use

Diagnosis, which we might define as the process or activity of identifying the nature and cause of a disease or injury, is central to both human and veterinary medicine. Yet, while the techniques and procedures of diagnosis have long attracted the attention of instructors and scholars, social science and sociological investigations of the practice of diagnosis have remained relatively few and far between (14, 15). This is particularly the case for the procedures and practices of veterinary diagnosis (16). Yet, recent developments in the social sciences themselves—drawing particularly on “more-than-human” studies and the Sociology of Science and Technology (16, 17) and also in the social sciences' attention to veterinary practices (18)–have raised the profile of veterinary diagnosis, its evolving social and professional context and its relationship to emerging technologies. As Hobson-West and Jutel argue [(16), p. 397] diagnosis confirms “the scientific and professional power of veterinarians” both with respect to animal owners and other para-professionals in the animal care sector.

At one level, the rising global concern for antimicrobial resistance and the role that farm animal veterinarians play in antimicrobial prescription and use brings a new attention to the processes, practices, and technologies of clinical diagnosis. However, the rapid and increasing use of antimicrobials throughout the latter half of the last century and beyond has raised specific issues and challenges for the role and place of veterinary diagnosis.

The arrival of antimicrobials in livestock systems, first in the US in the 1940s and then elsewhere (19), impacted significantly upon the traditional role and place of veterinary diagnostic practice within agriculture in two important ways. First, until partially restrictive legislation was finally introduced, both in the UK and in the US, low doses [defined as between 50 and 200 grams per ton of feed; Graham et al. (20) of commercially available antimicrobial drugs could be purchased legally and given to animals directly by farmers without the need for veterinary diagnosis, prescription or administration (21). There were many economic reasons for this, not the least being the dramatic upscaling of intensive livestock agriculture and the production of cheap and faster-growing animals, particularly poultry (22). Low dose, growth-promoting antimicrobials rapidly became an integral part not only of entire animal production systems but also of the structure of the consumer market for animal products. As Puig de la Bellacasa points out, “productionism colonizes all other relations” [(23), p. 184]. However, this also meant that in practice a growing proportion of antimicrobial use decisions were being made increasingly by farmers themselves and commercial advisors with little, if any, recourse to professional veterinary diagnostics. As H.C. Swann, writing in the British Veterinary Journal in 1963 reiterated:

When illness occurs in animals which a farmer suspects may be related to a certain system of feeding and possibly to the use of a particular foodstuff, he [the farmer] frequently seeks the free advice of the supplier's technical officer whose duty theoretically is to assess the situation in terms of the use of his firm's products, but it becomes increasingly difficult for him [the officer] to limit his advice to dietetics and husbandry. Consequently, free advice–not necessarily correct–is frequently given on disease problems by unqualified people (24).

Historically, farm animal veterinarians across the US (25), Germany (26), the UK (27, 28) and elsewhere in Europe (29) found themselves, albeit at different degrees, in an increasingly difficult position. With the expanding use of antimicrobials as growth promoters, easily obtained by farmers from feed suppliers and elsewhere and deployed without veterinary supervision (particularly in the rapidly intensifying poultry and later pig sectors), the capacity of veterinarians to control and manage antimicrobial use in agriculture was diminished. (30) argues that, in the 1950s and 1960s, many UK veterinarians and their organizations nevertheless became reconciled to the use of low dosage antimicrobials as growth promoters as a means of encouraging agricultural modernization, reducing animal feed costs and providing cheaper food to consumers, in the absence, at the time, of credible scientific evidence of the contribution of sub-therapeutic antimicrobial levels to both residuals and resistance transmission (25, 31, 32). Commenting on the relative failure of the 1969 Swann Report to address the issue of excessive antimicrobial use in agriculture, an editorial in the British Medical Journal in May 1980 claimed:

“… over-enthusiastic representatives of pharmaceutical firms as well as black market operators may find farmers, including poultry producers, all too ready to sidetrack their veterinarians and to bid for any supplies of prescription-only antibiotics that may become available through irregular channels. Prosecutions may close that door if evidence is forthcoming, and farmers need to be educated out of attempting to diagnose and treat or prevent enteritis by using antibiotics without veterinary help” (33).

In his incisive analysis of the later debates around the UK's Swann Committee and subsequent regulatory moves, Kirchhelle (27) suggests that the British veterinary profession, whose members both prescribed and sold antibiotics, sought control over antimicrobials “not only to generate income but to secure their profession's primacy over competing nutritional and health experts,” an observation that echoes (28) point that the UK veterinary profession's vision of the time was one that actively sought new areas of employment and income in response to a growing and intensifying livestock industry. Smith-Howard (34), writing on the US experience, has referred to this as a “leveling influence,” across the professions of farmer and veterinarian, but, as she points out, many within the veterinary professions of both the US and the UK saw the growing availability of antimicrobials to farmers as a direct challenge to the veterinarians' specific area of diagnostic expertise.

The second impact of the rapid introduction of antimicrobial use into the infrastructure of contemporary livestock farming on veterinary diagnostic practices has been the growth of preventative prophylactic and metaphylactic treatment. With the expanding intensification of livestock agriculture, animals were increasingly being treated with broad spectrum or “shotgun” antimicrobials administered premixed into animal feed both to stimulate growth but also to prevent subclinical disease. Indeed, as Kirchhelle has said:

“With antibiotic dosages in feeds increasing, the boundaries between growth promotion, prophylaxis and treatment soon blurred” (27).

Both in the UK and in the US, concern was being expressed as early as the 1960s that the “irrational” prophylactic prescription and use of antimicrobials to cure potential disease was making veterinary diagnosis irrelevant and largely redundant as a decision-making process across a range of endemic livestock diseases (24, 35). Within intensification regimes that were, to varying degrees, now established as the norm in many areas of livestock agriculture, antimicrobials were becoming not only, as Harrison called them in 1964 “a substitute for good husbandry,” they were also becoming a substitute for good diagnosis.

Commentators have seen the growth in prophylactic use of antimicrobials in livestock farming, often without veterinary diagnosis and on healthy animals (36), as a somewhat inevitable compensatory response to the halting or reduction of growth promotion in countries where such policies have been enacted (37). In a large number of European countries, providing antimicrobial medicines in the feed and water of farmed animals either to prevent disease (prophylaxis) or to halt the spread of disease already affecting one or more members of a group or flock (metaphylaxis) accounts for a greater proportion of total antimicrobial use in farming than the treatment of sick animals, particularly where they are regularly used to prevent disease in young piglets, poultry chicks and dairy cows (38–40). As Broll et al. (41) point out with respect to the prophylactic use of tetracyclines in German pig farms, such practices often occur without precise diagnosis and without confirmed disease presence. Moreover, in a number of circumstances, the medicines used in prophylactic treatment are considered critically important in human medicine (1) and may be deployed at far higher concentrations than were being used for growth promotion. In the UK, decreasing the use of antimicrobials for the prophylactic treatment of animals has become a major target in the drive to achieve more responsible and sustainable antimicrobial use in livestock farming (5, 42). This is in line with recent EU guidelines stating that “routine prophylaxis must be avoided” and that “prophylaxis should be reserved for exceptional case-specific indications” (43).

We might sum all this up by suggesting that the initial period of massive antimicrobial use in livestock agriculture, both for growth promotion and later for prophylactic treatment, has offered a challenge to the role and importance of veterinary diagnostics across a number of livestock systems, ironically at a time when diagnostic technology was improving considerably as procedures developed essentially for human medicine (such as those based upon the diagnostic potential of technologies such as polymerase chain reaction) were being transferred to veterinary medicine (44, 45). Highly effective, broad-spectrum antimicrobials engendered a widespread acceptance that the treatment is the diagnosis. These various challenges to veterinary diagnostics, it is claimed, not only helped contribute to what some argue has been an excessive and inappropriate use of such medicines in livestock farming (46), but also gave rise to the contested possibility of the proliferation of resistance on farms (47, 48).

## Practicing Veterinary Antimicrobial Stewardship

As the antimicrobial resistance “crisis” deepens and as governments and professional bodies mobilize to address and reduce unnecessary antimicrobial use in both human and animal populations, there has been a renewed emphasis on the role of farm animal veterinarians as critical players in the advocacy of antimicrobial stewardship. A term initially devised for human health care, “antimicrobial stewardship” has become widely adopted as a “as a set of coordinated interventions, as a programme, as a philosophy, and as an ethic” (49) across both human and animal contexts. Drawing on Landecker (50), it is now the problem of antimicrobial resistance rather than the antimicrobial solution to infectious disease that comes to define more recent biopolitical action. “As with antibiotics, the task of managing vitality turns to the control of the substances that were the previous technologies of production,” she writes (50).

Critically, the notion of antimicrobial stewardship combines, first, a more organizational and strategic path aimed at changing established practices, management contexts and long-term health care to allow for more sustainable and responsible antimicrobial use. As Page et al. (51) point out, this includes enhanced infection control, farm biosecurity, vaccination and on-farm health monitoring, all of which place the veterinary practitioner in a potentially different, more carefully negotiated, role with respect to farm clients, farm processes and farm technologies (52). A second path, informed by our survey and addressed below, is a more clinical and prescriptive path which includes the identification, selection, dosing, administration and duration of more specifically targeted and appropriate antimicrobial use for treating infection. In this second path, new practices and technologies of diagnostics and diagnostic testing become a key focus. Although both paths have arguably achieved a substantial reduction in antimicrobial use within the UK (53), the latter's emphasis on diagnosis, while, on the one hand, reaffirming the professional role and expertise of the veterinary clinician, raises, on the other hand, a number of issues within the context of antimicrobial stewardship and responsible medicine use, as we show below.

Looking in turn at these two paths, the first strategic component of antimicrobial stewardship clearly places specific responsibilities upon farm animal veterinarians. Here, the Swann Report of 1969, was prescient:

“We should like to see more use made of the veterinary surgeon as adviser when the introduction of an intensive enterprise is contemplated by a farmer so that disease may in some measure be prevented” (54).

In his 2009 review of veterinary expertise, Lowe referred to a sense of growing “marginalization” amongst the food animal veterinary profession in the UK, despite their critical position “not only between animals and their keepers, but also between government and farmers, between agriculture and the food industry and between the livestock sector and consumers” (11). This sense of marginalization had not been helped by repeated hesitations by the British government over past decades with respect to the introduction of a more State-supported preventative approach to on-farm disease management (28). Neither has it been helped by the profession's own difficult acceptance of the neoliberal shift to a more clientelist relationship with animal famers (12, 55) often in situations of growing competition with an emerging para-professional sector in farm animal health-care. In response, Lowe (11) has argued that veterinarians should take on a greater variety of problem-solving roles within livestock agriculture, moving away from a regulatory and purely clinical approach to more market-driven preventative roles with respect to animal disease and the food sector, an argument also taken up by Gardiner et al.

“The work of a dairy, poultry or pig specialist is not restricted (or even mainly focused) on consideration of the individual animal body; the “animal body” here is much more likely to be the whole herd or flock. The specialist role will incorporate a wide variety of management, preventive and agricultural economics issues, as well as attention to pressing public good issues such as animal welfare” (56).

This extended notion of the veterinarian's role is being increasingly adopted within the veterinary profession and recognized across a range of different political and professional cultures (57). The British Veterinary Association and the Royal College of Veterinary Surgeons joint publication “Vet Futures” (2015), for example, advocates greater involvement of the profession as a whole in wider issues of environmental sustainability, farm business planning, biosecurity and food health, alongside the more traditional fields of animal health and welfare. Furthermore, evidence from research confirms the growing importance of veterinarians as trusted suppliers of animal health, biosecurity and farm management advice and, in certain cases, the challenges they face in this new role (58–61).

Moreover, greater knowledge of resistance pathways on farms–derived from improved diagnostic testing along with closer pathogen and medicine monitoring–may allow veterinarians to prescribe antimicrobials not only to treat animal disease but, additionally, to reduce specific types of resistance across the entire farm through the notion of “cycling” antimicrobial treatments, an approach that puts veterinarians at the forefront of developing and extending better pharmacological understanding of both biosis and antibiosis (62).

This more holistic farm management approach to disease management offers considerable scope for innovative and interactive approaches to decision-making practices which foreground alternatives to more conventional mechanisms of external scientific expertise and regulatory authority. In dairy cattle, for instance, Morgans (63) argues that the relative success of both Danish and Dutch farmer groups in collaborating with veterinarians and scientists to achieve significantly reduced use of antimicrobials lies, in part, in the cooperative and facilitative nature of agricultural organization (64). In a recent paper, van Dijk et al. (65) report on the experimental establishment of a series of multi-actor participatory mechanisms enabling dairy farmers, veterinarians and food industry partners to collectively design and deliver practical on-farm changes to reduce antimicrobial use and maintain herd health and welfare. In both of these observations, we see not only shifts in both veterinary and husbandry practice but also part of a more fundamental reassessment of the ways in which herd health planning and disease management is addressed in livestock production. It is increasingly asserted that such responses are having an impact. Longer weaning times, better health management of groups of animals, vaccination, enriched housing, better ventilation and temperature control, the separation of ill animals from the herd/group, the use of slower growing breeds and lower stocking densities and many more all contribute to reducing the need for antimicrobial treatments (5, 66–68).

Annual audits confirm that the total volumes of antimicrobials used in livestock agriculture in the UK are falling (53, 69, 70). Between 2012 and 2018, the total amount of Critically Important Antimicrobials (CIA) purchased, prescribed and/or administered in a survey covering 90% of the UK broiler sector (including ducks and turkeys) fell by 82.6% (71). In the UK pig sector, recent data from the Veterinary Medicines Directorate reveals a 60% fall in antibiotic usage between 2015 and 2018 (53). While some commentators have suggested that these falls represent the ‘low hanging fruit' of antimicrobial use reduction, they nonetheless demonstrate that significant achievements can already be attained through improved stewardship, and disease management.

## Rapid and Point-Of-Care Diagnostics

The emerging resistance “crisis” of the last ten or so years–largely in human medicine but also to a lesser extent in animal health–has considerably refocused debate on the second, more clinical, pathway of antimicrobial stewardship and, in particular, on the technologies and practices of diagnosis. Coupled with this has been the growing emphasis on “evidence-based” medical and veterinary practice, which in turn places renewed attention on diagnosis as well as on parameters such as the sensitivity and specificity of individual test procedures (72). Within the wider debate on diagnostic tools and tests, the last decade has also seen a considerable emphasis on rapid or point-of-care tests as having a particular contribution to make in reducing unnecessary antimicrobial use. Acknowledging that “diagnostics have had less impact on antimicrobial prescribing than might have been expected,” the Wellcome Trust argued in 2016 that:

“Rapid diagnostics are thought to have a vital role to play in the battle against drug- resistant infections. They have the potential to guide more rational use of antibiotics, by distinguishing between viral and bacterial infections, and by identifying specific pathogens and their antibiotic resistance characteristics” (73).

Returning to the final report of the O'Neill review of 2016, we find a similar emphasis on the use of diagnostics:

“Fundamental change is required in the way that antibiotics are consumed and prescribed, to preserve the usefulness of existing products for longer and to reduce the urgency of discovering new ones. Rapid point-of-care diagnostic tests are a central part of the solution to this demand problem, which results currently in enormous unnecessary antibiotic use” (3).

The reasons for this new focus on diagnosis and diagnostic tests are essentially three-fold, based around issues of technological development, rapidity, and evidence. While significant advances in the technologies of diagnostic testing for many infectious diseases (74) have allowed testing technologies to become not only smaller and more portable (75), making them more accessible to individual and corporate veterinary practices, rapid and point-of-care tests are not available for all aspects of diagnosis relevant to antimicrobial selection. Conventional reliance on the traditional “pipeline” –where samples are sent to centralized laboratories and test results communicated to veterinarians—can be a hindrance to urgent treatment decision making. Newer diagnostic technologies could offer, it is claimed, more rapid results (76), accurately and at lower cost (77). Finally, as the drive toward more evidence-based veterinary medicine gains strength, and as concern grows over the inappropriate use of antimicrobials to treat animal disease, we note an increasing attention being paid to diagnostic tests as critical legitimators of treatment decisions, particularly with respect to the use of antimicrobials. This can be seen in the expanding use by assurance and certification schemes of mandatory diagnostic tests for the deployment of critically important antimicrobials (78).

While recognizing the importance and potential of rapid and point-of-care diagnostics in contributing to reductions in antimicrobial use, many commentators–from science, policy, and industry–also acknowledge that significant economic, institutional, and practical barriers exist in bringing newly developed rapid tests to the marketplace (1, 79, 80). Various initiatives, such as the UK Longitude prize (awarded for the development of new point-of-care diagnostic tests in human medicine), are seeking to address these barriers through the offer of specific stimulus to new diagnostic development (Longitude.org, undated). Our concern in the remainder of this paper, however, is not what hinders the emergence of new rapid and point-of-care diagnostic tests but rather the manner in which such tests are both currently used and anticipated in clinical farm animal veterinary practice and the possible impact rapid test technologies might have on broader farm animal health management and disease control.

## Methods

A qualitative, semi-structured interview-based survey (81) was undertaken of 30 farm animal veterinarians currently working in farm animal clinical practice and drawing evenly across the poultry, dairy and pig sectors in England, Scotland, and Wales. The aim of the interviews–drawing in part on an earlier study (82)–was to explore veterinary roles in active antimicrobial stewardship, first, by investigating current diagnostic practices–particularly within the context of antimicrobial prescription and use–and, second, by exploring with veterinarians the current deployment and future impact of rapid and point-of-care diagnostic tests in contributing to more selective and reduced use of antimicrobials. The interviews were divided into four thematic foci addressing in turn: recent employment history; current diagnostic practices (employing “walk through” and narrative accounts); current experience with rapid and point-of-care diagnostic tests; and the relationship between diagnostic practice and antimicrobial prescription and use. Interviews were carried out across a variety of regions of Great Britain, ranging from South West England to Scotland and reflecting geographical concentrations of the three main production types (dairy, pigs, and poultry). The sample was generated initially through contacts with partner veterinary practices and later extended through snowballing techniques, personal contacts and, in the pig and poultry sectors where numbers of veterinarians are substantially smaller, through targeted solicitation. Further details on the interviewees are provided in Table 1. All interviews were carried out in 2019, the majority in the first three months of the year.

**Table 1:** Veterinarians interviewed with practice type, gender, and location.

**Interviewee**	**Practice**	**Location**
10 cattle veterinarians	6 from corporate practices 4 from independent practices 5 males and 5 females	The South West of England, East Midlands, and East Anglia
9 poultry veterinarians and 1 poultry welfare consultant	3 from corporate practices 5 from independent practices 2 consultant veterinarians 8 males and 2 females	Scotland, South West England, East Anglia, East Midlands, Yorks/Humber, South East England, and the North West
10 pig veterinarians	3 consultant veterinarians, 6 from independent practices 1 food company veterinarian 4 males and 6 females	Wales, South West England, East Anglia, Yorks/Humber, and Scotland

*Source: Authors Survey, 2019*.

The veterinarians ranged in age and experience from recent graduates to veterinary practice directors with many years' experience. Of the interviewees, 13/30 were female. Most interviewees worked at independent veterinary practices (though many of these had multi-site offices) and a few were either employed by larger corporate veterinary companies or acted as independent veterinary consultants. The majority of the interviews were carried out face-to-face at the veterinary practice (23 of the 30, with seven being undertaken on “Skype”) and were, in each case, undertaken by two interviewers drawing across the social and the veterinary sciences. Interviews lasted between one and two hours and were recorded, anonymized and transcribed. Ethical approval for the survey was granted by the University of Exeter Geography Ethics Committee, approval reference number eCLESGeo000069v.3.0.

Analysis of the interview transcripts was initially undertaken through standard systematic thematic coding techniques, allowing a series of common themes to emerge from the responses (83). A more detailed analysis of the three sets of interview transcripts (pig, poultry, and dairy cattle) was undertaken by the research team drawing on the Realistic Evaluation method, originally developed by Pawson and Tilly (84) and its emphasis on Context, Mechanisms, and Outcomes (85). For the purposes of this paper, the analysis focused more specifically upon interviewee responses to the third and fourth foci of the interviews: experience and use of rapid and point-of-care diagnostic tests and the relationship of diagnostic testing to antimicrobial stewardship. From that analysis, three particular inter-related themes emerged from the interview transcripts: first, how the use of rapid and point-of-care diagnostic tests challenges established practices and assumptions on the part of veterinarians; second, how rapid tests, while useful in many contexts (particularly within dairy farming) run up against the complex disease etiology of more intensively farmed species such as pigs and poultry and; third, how the growing commercial availability of rapid and point-of-care diagnostic tests is leading to a diversification of responsibilities and actions in disease monitoring, assessment and treatment, with implications both for the professional role of clinical veterinarians and for the responsible stewardship of antimicrobial use. Taken together, these three themes provide insight into how the future development and deployment of rapid and point-of-care diagnostics might articulate with the practices and concerns of clinical veterinarians working in different sectors of livestock production.

## Results and Discussion

As we have shown above, rapid and point-of-care diagnostic tests are widely seen within various policy and scientific communities as part of the “solution” to excessive antimicrobial use in both human and animal health care: “a step change in the way that technology is incorporated into the decision-making process around antibiotic use” (1). In their adoption by clinical veterinarians across different production sectors (here, dairy, pig, and poultry) and across different animal health conditions, rapid and point-of-care diagnostic practices introduce or expose new and different levels of complexity, be they in the test parameters and on-farm sampling environment, the animals themselves (their multiple biomes and their pre-existing conditions and treatments) or in the very divisions of labor that characterize the performance of diagnostic testing. Certainly, both the approach to, and general usage of, rapid and point-of care diagnostics varies significantly across the three production sectors as do the levels of complexity raised by that usage. The following section draws out those variations but is also attentive to the more common issues, across all three sectors, that are raised by rapid and point-of-care diagnostic tests, widely seen, as cited above, as a generic “solution” to inappropriate antimicrobial use in livestock farming.

### The Practices of On-Farm Rapid Diagnostic Testing

Rapid and point-of-care diagnostic tests can be carried out on-farm with acceptable sensitivity and specificity, producing fast results that allow treatment decisions to be made quickly, accurately and at lower cost (77). Yet, at the present time, their availability is relatively limited within clinical farm animal veterinary practice and varies significantly across production sectors. In both the commercial (and highly integrated) poultry and pig sectors, where health management and treatment take place largely through regular vaccinations and feed or water-based medication, through an attentiveness to the microbiome of group housing and where staged interventions are generally at a group or flock level rather than at the level of individual animals, we found rapid and point-of-care diagnostic tests to be currently less common than in the dairy sector.

Poultry veterinarians interviewed in the course of this research stated that the most frequently used on-site diagnostic tests were conducted post-mortem, as its relative inexpensiveness rendered other diagnostic tests less valuable in the health management of individual fast-growing flocks with limited treatment opportunities. The poultry veterinarians nevertheless saw the potential for new rapid, on-farm testing to contribute to more specifically targeted antimicrobial use but recognized that the tests were simply not available at the current time. One possible technological development of interest to poultry veterinarians was portable PCR machines:

“They've been looking at PCR-type tests that you can take onto farms that will tell you from your swab whether it was *E. coli* or whether it was *Pasteurella* or something like that. That would be very useful. Yes, we expect it to be *E. coli*. It would be even better if we could turn around and say, ‘This *E. coli* that you've got in there is actually likely to be pathogenic, as opposed to just a post-mortem contaminant'” (DIAL Project: Poultry veterinarian P.03a.19).

Similarly, pig veterinarians, operating in a sector that is substantively different from either poultry or dairy, with disease management at group level characterized by a strong emphasis on broader epidemiological approaches and assessment of on-farm bacteriological and resistance histories, were interested in speeding up the testing results. In the words of one pig veterinarian: “I think the biggest problem we have is that rapidity is not there” (DIAL Project, Pig veterinarian 29/19). Pig veterinarians pointed to the benefits of a rapid test that would be able to differentiate specific pathogens and diseases such as *Streptococcus suis* meningitis and bowel edema in weaners caused by strains of *E. coli*, where post-mortem examinations yield insufficiently precise results. For some, the advantages of rapid tests were straightforward:

“If you could plop a drop of scour [diarrhea] on a plate it says: Yes it's an *E. coli*. Yes it has virulence factors such like for you to be significant and this is its resistance profile, then that would allow you to institute treatment faster and more logically” (DIAL Project, Pig veterinarian Pg.03.19).

Dairy cattle veterinarians, however, given both the value and relative importance of individual animals as milk producers as well as the more diverse structure of the industry itself, used rapid and point-of-care diagnostic tests far more commonly than veterinarians in the pig and poultry sectors. A number of well-known and widely available commercial tests exist for dairy cattle veterinarians, though not all would necessary be deployed in the case of identifying the possible need for an antimicrobial treatment. Nevertheless, here too the more general adoption of rapid tests was nonetheless limited by a number of factors (86), including (1) concerns over the practical use on rapid tests in on-farm situations, (2) their limited range of disease applications and (3) by the fact that, for many cattle veterinarians, these tests rarely offer unequivocal confirmation of a specific pathogen on which to base the prescription of antimicrobials. Indeed, perceptions of the accuracy of rapid and point-of-care tests was a theme that emerged repeatedly in our survey with a number of veterinarians maintaining that manufacturers' sensitivity and specificity specifications were not always easy to ascertain for rapid tests. As one cattle veterinarian put it:

“I'm not sure of the sensitivity of those things, so even in the absence of a bacterial positive, I'm not sure we'd always be brave enough to say we're not going to treat on that. I think the rapidity depends on the aspect of it. It can sensibly limit the benefit of those so if they don't have a sensitivity that's as good as the lab's, or nearly as good, then they're probably not going to be used then. The largest convenience factor is it's quite easy just to run that through there” (DIAL Project, Cattle veterinarian C.05.19).

In the balance between convenience and accuracy, veterinarians confirm that they sometimes intentionally privileged the former when it came to the use of rapid tests. The decision to use a rapid or point-of-care test was frequently driven by the need to provide the client with an immediate answer.

“I've heard people say before that maybe it's less sensitive but if they could prove the sensitivity of it… To be honest I think a farmer would rather have an answer even if you say “Look, there's maybe like even 20% chance that this could be a false positive,” I think they'd rather have that 80% chance that it is correct, if you see what I mean. I know with tests they've got to have the really high specificity and sensitivity but actually the farmer doesn't really care about that. As long as it's an overall majority he would rather take a hit at that than have nothing at all” (DIAL Project, Cattle veterinarian C.08.19).

Veterinarian concern for how notification of disease presence might be taken by the farmer seemed to implicitly raise the value, for some, of “scientific evidence,” as justifying the necessity of treatment or confidence not to treat. Here, the act of scientific diagnosis and the exclusive role of veterinarians in performing diagnosis (and subsequently prescribing treatment) becomes a key component of professional legitimation.

“There's no getting around the fact that if you can present someone with the scientific evidence like they've definitely got this disease and it's definitely a problem and it's definitely sensitive to this then you've got a far better case for treating it then being like, “Well, I think 70% sure it's got this but it will take me three weeks to confirm it so I've just guessed because I can't really…” like that's always going to be a weaker case for antibiotic use” (DIAL Project, Poultry veterinarian, P.03b.19).

Where sensitivity and specificity are important to the diagnostic decision, in the eyes of veterinarians across all three sectors, laboratories remain the standard reference even though the cost is often higher, the time taken to receive the results longer and any limitations to the test are identifiable. As another cattle veterinarian remarked:

“I try and be as rigorous as possible and the labs now are very good–they always offer that element, certainly when it comes to testing for infectious diseases on antibody levels, now the labs will always put the specificity, sensitivity of the test at the bottom of your results, so that is extremely helpful and very good practice. I think it's just a very good reminder that there are limitations to the test” (DIAL Project, Cattle veterinarian C.03a.19).

Although veterinarians across the three sectors maintained that there remained a gap in the market for rapid and point-of-care diagnostic tests, it is significant that many saw the future of such devices not in specifically reducing unnecessary antimicrobial use but as part of an extended diagnostic pathway that might begin with a simple “rule-in” or “rule out” and then lead to a more investigative testing or treatment: “A positive result, it's easy isn't it? It's the negative that is difficult” (DIAL Project, Cattle veterinarian); “Pen side tests are great but […] it just then prompts you to investigate a bit further” (DIAL Project, Pig veterinarian). When compared with laboratory tests–which frequently include a range of test parameters as well as predictive values, sensitivity and specificity information and which are getting faster in their delivery–rapid tests, in the words of one interviewed pig veterinarian: “will not give you enough information to treat well”. Veterinarians interviewed in the course of this research stated that what would be most useful to them would be a simple test that distinguished a bacterial from a viral infection while accepting that this would only ever be a diagnostic starting point. What remains abundantly clear, however, is that rapid or point-of-care tests are not seen by UK livestock veterinarians, at least at the current time, as the critical panacea for antimicrobial use reduction.

### Animal Complexities

The relative simplifications inherent in rapid and point-of-care diagnostic tests, though seen as a considerable advantage in speed and potential pathogen or disease targeting, also came up against the complexity of on-farm biotic environments and the multispecies “messmates” (87) that co-constitute farmed animal bodies. Acknowledging the complex biome of pig production units and the difficulties of pinpointing the precise cause of an illness, pig veterinarians expressed certain reservations over the possibility of widescale adoption of current rapid and point-of-care diagnostic tests as definitive mechanisms for identifying pathogens and disease:

“Pneumonias and things like that, you do get outbreaks of and cause problems. I don't know if they'd be more difficult to do as a rapid test because I suppose you do them more on the nasal swabs and things like that because your … serology on your respiratory ones are a little bit less reliable in terms of it's not showing that's what's causing the problem and you probably have various things involved and it's a bit more—yes I'm not sure how well you could rely on that test. […] There's a bit of this but they are positive for that antibody-wise but is there something else involved as well” (DIAL Project: Pig veterinarian Pg.02.19).

Other veterinarians pointed to the difficulties of securing a relevant test result in the face of pre-existing treatments:

“We've used the vaccine, we create the test so then we can make a diagnosis, but with difficulty. Now we've got easy tests to use we can't make the diagnosis because we've got too much vaccine” (DIAL Project: Poultry veterinarian P.03a.19).

The focus—or reliance–upon the presence or absence of particular markers, on changing strips of color or on positive or negative read-outs is a perhaps a necessary but nonetheless problematic simplification. Farms are complex microbial spaces with every farm displaying a unique mix of pathogens, many of which might be endemic and/or subclinical but nonetheless present.

There's a lot of diseases that we have problems with […] that cause a lot of sub-clinical disease that we do diagnose but because we vaccinate with live vaccines snap tests aren't massively useful for that and that could trigger off quite a few diseases, like we vaccinate but our vaccines can only do so much so we quite often get sub-clinical disease (DIAL project, poultry veterinarian P.03b.19).

Infections with some pathogens may be, to some extent, obscured by other infections–viral or otherwise–leading to complex disease etiologies. Individual animals, herds, or flocks may exhibit temporary microbial imbalances or dysbiosis, particularly within the confined and complicated microbiome of the contemporary industrial farm. The very diversity and ubiquity of microbes within larger organisms–such as farmed animals–and their groups, challenges the notion of simple disease presence or absence and suggests that diseases emerge from highly complex and, in many cases, potentially beneficial “pathobiomes” (88), themselves highly contingent upon the material spaces, the microbial histories and the contemporary management practices of farms. It is in the ability to distinguish and select amongst this complex environment that the broader “empirical” diagnostic practice reveals its greater value. This perhaps suggests that any simplification of diagnostic processes may well have a localized impact on reducing some erroneous prescription and treatment decisions but may do little, if anything, to manage the wider biotic complexity of the contemporary farm.

### Diversifying Animal Health Responsibilities

The role and place of diagnostic testing in farm animal clinical veterinary practice is changing. In part, this is in recognition of concerns over the inappropriate and excessive use of antimicrobials in livestock production systems (82, 89–91)]. Many former practices of herd- or flock-level prophylactic or metaphylactic treatment with critically important antimicrobials are disappearing, certainly in the UK.

“It used to be terrible. Don't get me wrong, we used to put chlortetracycline in feed for every crop of broilers, we didn't care, we'd do three days of enrofloxacin at the start of crop just in case there were any issues” (DIAL Project: Poultry veterinarian P.03b.19).

However, it is far from clear from this research the extent to which, after the O'Neill report, rapid and point-of care technologies will contribute specifically to further reductions in antimicrobial prescription and use. Ironically perhaps, the singular results of rapid and point-of-care tests often require greater interpretation and judgement on the part of the skilled veterinarian than more complex multi-parameter lab-based testing from which a more informed picture of disease etiology can be built. This too raises issues of consistency in both the interpretation of rapid test results and subsequent treatment decisions.

One particular consequence of the growth of rapid and point-of-care diagnostic tests that we draw from the current research is the impact upon the extension and diversification of responsibilities for animal health. The move from purpose-built laboratories to veterinary practice labs as a location of testing and now potentially to rapid on-farm diagnostics alters the accessibility of these different technologies. Many rapid diagnostic tests can be purchased commercially in farm service outlets and are increasingly being used by farmers themselves, generally (but not always) under veterinary supervision, not only to monitor animal health but also as the basis for subsequent treatment decisions. This has implications, not only for the traditional authority and role of the veterinarian, but also for the nature of the treatment decisions that follow. As one cattle veterinarian put it:

“There's a fear with *[named on-farm culture test]* on the farms I work with, [farmers] are under a lot of pressure to become a technician, become involved in making the decisions. […] However, I think within a few weeks I'd be cut out the loop and he [the farmer] would be the diagnoser and the treatment instigator and making bad decisions and all that” (DIAL Project, Cattle veterinarian C.02a.19).

For many farm animal veterinarians, this diversification of roles and responsibilities is welcome and many practices have developed (frequently laminated) protocols for client farmers to follow in sampling, in testing, in interpreting the results and in making subsequent treatment decisions, usually in accordance with a pre-determined health plan; for example, the use of culture plates in mastitis management.

“We would go out and train them in what to do and then we'd get them to do it. Then the question is three weeks later when they're doing it on their own, are they still doing it properly or have they forgotten or cut corners, or did they forget to switch the incubator on or whatever? All the things that can go wrong, and some farmers obviously are very much better at following protocols than others and some farmers just have this desire to just do their own thing all the time” (DIAL Project, Cattle veterinarian C.02b.19).

Regular animal health monitoring by farmers and farm staff can also build up a more complete picture of herd and flock health, an obvious advantage when veterinary interventions are required. Some veterinarians, however, expressed discontent about the risks associated with such role diversification and diagnostic simplification. These included contamination of samples, poor maintenance of the test environment, misinterpretation of the results and, as a consequence of any of these, unnecessary or inappropriate medicine use. The use of the formalized and pre-determined “protocol” thus becomes critical in the legitimation of veterinary authority when tests are being used by farmers.

“It makes me nervous … because at the end of the day I am responsible for prescribing on that farm. If they're doing a test and they're making a choice about what they use, I stopped being relevant to that decision-making process. Whereas if we had a set protocol–this is what you do–I've kind of okayed it, they know the parameters within which they can operate, and we know what it needs” (DIAL Project, Cattle veterinarian C.03a.19).

As the technologies of animal health monitoring and surveillance become more portable, faster and easier to operate, this diversification of roles may stretch beyond the farmer to include even broader food chain actors. Farms, particularly those firmly integrated into vertical chains, are increasingly monitored through rapid technologies, whether by milk companies, food processors, assurance, and certification schemes or food retailers. This clearly has implications for the veterinarian's clinical role as the following three comments from interviewed veterinarians demonstrate:

“I must say that it seems to me that in this country because there are other bodies that are very heavily involved with monitoring milk by different aspects it's actually not easy to get a grip of what goes on when it comes to not just milk quality but also very much the bacteriological aspect of milk. There seems to be a little bit of a dichotomy to be honest and vets seem to be a little bit… you really need to be proactive otherwise nobody really offers [the monitoring data]” (DIAL Project, Cattle veterinarian C.03b.19).

“Tests [as opposed to diagnostics] are increasing ordered by food chain actors—vets not involved; animals are being slaughtered or treated unnecessarily” (DIAL Project, Cattle veterinarian C.02b.19).

“I think our whole business model is changing and has got to change. If we take, for example, the poultry sector, the vet is an advisor, at the end of the day; they will be doing some tests on the farm, all the farm post-mortems or whatever. They're capturing data that's fed back up the chain and becomes relevant to management decisions up there” (DIAL Project: cattle veterinarian C.03a.19).

Unsurprisingly perhaps in the face of this growing diversification of roles, a counter tendency is observable that seeks to reinforce and strengthen the very specific legitimation and conventionalization of veterinary authority. Under the UK Veterinary Surgeons Act (1966), diagnosis can only legally be carried out by veterinarians. This unique authority is specifically and newly mobilized in the insistence–first by the Red Tractor Assurance scheme and now followed by others–that critically important antimicrobials can only be used on assured farms when preceded by a diagnostic test undertaken by a veterinarian (78). However, as non-veterinary practitioner sources of advice, data and services relating to animal health and disease management continue to multiply (60), the boundaries of what constitutes a diagnosis are becoming less and less clearly defined, and veterinarians can no longer rely solely on such traditional Aesculapian authority awarded uniquely to those who heal (92).

## Conclusion

Diagnosis, as a practice, as a form of specific expertise, as both a scientific and a social process lies at the very center of medical and veterinary activity and professional legitimacy. Yet diagnostic practice is changing.

Diagnoses are […] contested, socially created, framed and/or enacted. And while diagnosis of disease is “central to the practice of medicine” as Blaxter put it [2009] and as the context of the practice of medicine has changed, so too has the play of social, political, technological, cultural, and economic forces which impinge upon diagnostic categories and diagnostic processes [(93), p. 793].

In this paper, we have demonstrated how, in their responses to the requirements of antimicrobial stewardship and, in particular, to the growing policy emphasis being placed upon rapid and point-of-care diagnostic test technologies, farm animal veterinarians interrogate and respond to the shifting context of diagnostic testing, revealing what is today a complex and by no means singular landscape of practice. While veterinarians share concerns around the diagnostic power and potential of currently available and developing rapid and point-of-care tests compared with laboratory test procedures–especially when antimicrobial prescriptions might follow—expediency and rapidity within the context of veterinarian/client relations emerges as a strong driver for the expanding use of these technologies, particularly, but not exclusively, within the dairy sector. Increasingly common calls from veterinarians for a simplified, cheap and accessible method to differentiate viral from bacterial infections on-farm are, to a degree, mitigated by veterinary experience that disease interactions are often highly intricate, multiple, and inter-related. In such instances, rapid and point-of care tests might offer, in certain conditions, what might be considered a problematic simplification of diagnostic practice.

The diversification and potential multiplication of test practices and health monitoring mechanisms that do not require a veterinarian and are becoming widely facilitated by rapid and point-of-care technologies emerge from this study as something of a double-edged sword. While veterinarians might welcome the additional data and information that testing undertaken by farm staff and other food chain actors might bring (as long as it is made available to them and can be used holistically), veterinarians also express concern over the conditions, accuracy and interpretation of the test results by non-clinicians which inevitably complicates the veterinarian's traditional responsibility and authority for diagnosis and animal treatment. What nevertheless remains clear is that rapid or point-of-care tests are not seen by UK farm animal veterinarians, at least at the current time, as the critical panacea for antimicrobial use reduction across all production sectors.

## Data Availability Statement

The datasets generated for this article are not readily available because the data set will be deposited in UKRI depository at termination of contracted research. Requests to access the datasets should be directed to https://www.dialamr.com.

## Ethics Statement

The studies involving human participants were reviewed and approved by University of Exeter, Geography Ethics Committee. The patients/participants provided their written informed consent to participate in this study.

## Author Contributions

HB, KR, ABr, and SH led the conception and design of the study with contributions from KC, LM, ABa, KA, and GR. The data collection was carried out by KC, LM, GR, and KA. Analysis of the data was undertaken by HB, KC, and SH with contributions from KR, GR, LM, ABa, ABr, and KA. HB wrote the first draft of the manuscript. KR, KC, SH, ABr, ABa, GR, LM, HB, and KA contributed to the manuscript revision and interpretation of the data and all authors have read the paper.

## Conflict of Interest

The authors declare that the research was conducted in the absence of any commercial or financial relationships that could be construed as a potential conflict of interest.
